# Evaluation of the Effect of Cellular Phones on Salivary Levels of IL-10

**Published:** 2019-02

**Authors:** Ali PEDRAMI, Fateme ARBABI-KALATI

**Affiliations:** 1. Department of Oral Medicine, Zahedan University of Medical Sciences, Zahedan, Iran; 2. Department of Oral Medicine, Oral and Dental Disease Research Center, Zahedan University of Medical Sciences, Zahedan, Iran

## Dear Editor-in-Chief

Currently, billion cellular phones are in use all over the world. During the last decade use of cellular phones has increased steeply, becoming a cultured tool(1). One major concern is the possible detrimental effects of electromagnetic waves and radio frequencies and macrowaves from cellular phones and base transceiver stations (BTSs) on health(2).

Although there are contradictory reports on the tumorigenic effects of non-ionizing radiations of cellular phones, electromagnetic waves are classified as possible oncogenic agents for humans (3). Various studies have yielded contradictory results about the effects of cellular phones on the chemical composition of the saliva and its antioxidative system; however, only one study has evaluated the effects of cellular phones on the inflammatory components of the saliva (4).Use of cellular phones results in a decrease in salivary IL-10 levels and an increase in IL-1β levels. IL-1β is a proinflammatory cytokine and IL-10 is an anti-inflammatory cytokine. Use of cellular phones results in changes in theprofile of salivary cytokines, exacerbating inflammation. However, inadequate data are available on the subject at present, necessitating further investigations.

Forty deaf volunteers were selected as a control group. Then 40 subjects were selected, on a voluntary basis, from the patients referring to the Department of Oral Medicine, Zahedan University of Medical Sciences, Zahedan, Iran in 2016–2017, who had a history of the use of cellular phones for more than 5 yr with daily conversations on the phone for more than 20 min andless than 60 min. The two groups were matched in terms of age and gender.

The protocol of the present study was approved by the Ethics Committee of Zahedan University of Medical Sciences. Informed consent was obtained from all the participants

Spitting technique was used to collect salivary samples. Each subject was asked to collect his/her sample in a 15-mL Falcon tube for 2 min. Then each tube containing the salivary sample was coded and immediately centrifugedat 2500 rpmto separate possible debris. Then the pure salivary samples were stored at −70°C until they were tested. All the samples were collected from 9 to 11 in the morning.

In order to examine the amount of salivary IL 10 levels, enzyme-linked immunosorbent assay (ELI-SA) method was used (R&D, Italy).Data were analyzed with independent t-test using SPSS ver. 21 (Chicago, IL, USA).

The mean ages of the subjects in the case and control groups were 25.53±2.7 and 27.09±4.8 yr, respectively. The mean salivary levels of IL-10 for case and control group was 139.70±66.39 and 72.68±38.68, respectively (*P*<0.001). There was significant difference between the two groups and salivary IL 10 was higher in case group.

The results of the present study showed a higher level of whole saliva IL-10 levels in cellular phone users compared to deaf subjects as a control group.

Thereare limited studiesavailable on the effects of cellular phones or electromagnetic waves on IL-10 levels.Useof cellular phones resulted in a decrease in IL-10 levels of saliva secreted by parotid glands in the vicinity of cellular phones, which is different from the results of the present study. The saliva secreted by the parotid gland adjacent to the cellular phone was compared with the contralateral side; however, in the present study, IL-10 levels were evaluated in whole saliva. On the other hand, salivary IL-10 levels had decreased to a greater degree in patients used cellular phones for more than 10 yr. However, in the present study, the subjects had a history of cellular phone use for 5 years. The effects of cellular phones on salivary cytokines change over time (4).

It is yet to be clarified whether or not a change in the salivary cytokine levels of cellular phone users is associated with tumorigenesis, and further long-term studies are necessary for this respect. Living near a BTS leads to some conditions such as headaches, sleep disorders, depression, anxiety, lack of concentration and anorexia. Long-term use of cellular phones might result in similar effects. On the other hand, salivary IL-10 levels increase in response to stress, which might explain the increase in salivary IL-10 levels in the present study(5).

However, use of cellular phones might affect the immune system of the saliva.
